# Conv-Former: A Novel Network Combining Convolution and Self-Attention for Image Quality Assessment

**DOI:** 10.3390/s23010427

**Published:** 2022-12-30

**Authors:** Lintao Han, Hengyi Lv, Yuchen Zhao, Hailong Liu, Guoling Bi, Zhiyong Yin, Yuqiang Fang

**Affiliations:** 1 Changchun Institute of Optics, Fine Mechanics and Physics, Chinese Academy of Sciences, Changchun 130033, China; 2 College of Materials Science and Opto-Electronic Technology, University of Chinese Academy of Sciences, Beijing 100049, China; 3 Department of Electrical and Optical Engineering, Space Engineering University, Beijing 101416, China

**Keywords:** image quality assessment, vision transformer, self-attention, neural network, deep model interation

## Abstract

To address the challenge of no-reference image quality assessment (NR-IQA) for authentically and synthetically distorted images, we propose a novel network called the Combining Convolution and Self-Attention for Image Quality Assessment network (Conv-Former). Our model uses a multi-stage transformer architecture similar to that of ResNet-50 to represent appropriate perceptual mechanisms in image quality assessment (IQA) to build an accurate IQA model. We employ adaptive learnable position embedding to handle images with arbitrary resolution. We propose a new transformer block (TB) by taking advantage of transformers to capture long-range dependencies, and of local information perception (LIP) to model local features for enhanced representation learning. The module increases the model’s understanding of the image content. Dual path pooling (DPP) is used to keep more contextual image quality information in feature downsampling. Experimental results verify that Conv-Former not only outperforms the state-of-the-art methods on authentic image databases, but also achieves competing performances on synthetic image databases which demonstrate the strong fitting performance and generalization capability of our proposed model.

## 1. Introduction

Image quality assessment (IQA) is a branch of computer vision research that aims to give computers the same ability to judge image quality as humans. IQA is a crucial research area since it can be applied to image restoration and used to distinguish between distinct visual perception experiences [1]. These days, digital images captured by cameras on cell phones, professional imaging equipment, and remote sensing satellites are widely used [2]. Digital images introduce noise signals in the acquisition process, and they can lose perceptual information during compression, storage, and transmission [3]. As a result, IQA is becoming increasingly important and has numerous applications, such as designing image restoration models to remove blur, noise, clouds, and other artifacts from images using IQA algorithms. It can also assist image acquisition equipment in evaluating and debugging the product’s imaging parameters and determining whether the imaging system is degraded. Although subjective human assessment can truly reflect human visual perception, and the assessment results are direct, accurate, and reliable, which is the ultimate basis for judging image quality, the implementation process is time-consuming and costly. It is also easily influenced by personal subjective emotions and preferences, and it has many limitations in meeting practical application needs [4]. To promote the use of IQA in real-world engineering, accurate and effective objective IQA algorithms must be developed.

The objective IQA method is an evaluation method that builds a mathematical model based on the visual system of the human eye and scores the image to be measured. This method is low-cost, has the advantages of batch processing and reproducible results, and can be more easily applied to a variety of scenarios. Traditionally, objective IQA algorithms are classified into three categories based on whether a reference image is required or not: full reference IQA (FR-IQA) [5], reduced-reference IQA (RR-IQA) [6], and no reference IQA (NR-IQA) [7]. The full-reference image quality evaluation method uses the image under test to compare with the original reference image in an adequately defined image space; the reduced-reference image quality evaluation does not use the original image directly but uses some information of the original image such as structural features and distortion types to evaluate the image quality, and the no-reference image quality evaluation does not use any original reference image information in the evaluation process. The main goal of all three image quality evaluation methods is to predict a quality score relevant to human visual perception. NR-IQA has attracted a large number of researchers’ attention in recent years because no reference information is available or may not even exist in many realistic situations. NR-IQA has been a challenging problem that has not been well addressed by many methods.

Early models for NR-IQA were based on manual feature extraction [8,9,10], which relied heavily on our summarized knowledge of the probabilistic architecture of the visual world, the mechanisms of image degradation, and the composition of the human visual system (HVS). In recent years with the development of convolutional neural networks (CNN), more and more vision tasks have benefited from this [11,12,13]. Current deep learning-based image quality assessment methods have achieved remarkable success in extracting visual features using CNN [4,14], which have the following advantages over manual feature extraction:

(1) By carefully designing a deep neural network with the problem to be solved and the input data, it is possible to automatically learn the relationships implicit within the data from the training dataset without the need for tedious manual feature extraction;
(2) Deep neural network models can contain thousands of parameters; thus, deep features can have better differentiation and representation capabilities. Compared with manually extracted features, it has more prominent advantages in extracting multi-level features and contextual information of images;
(3) Deep learning can change the model architecture by simply adjusting the parameters, which enables the network to automatically model itself according to the specific characteristics of the task, with good generalization and efficiency.

In recent years, Self-Attention based transformer architecture has achieved great success in the field of natural language processing (NLP) by establishing a long-range interaction in a scalable manner [15,16,17,18] and continues to make constant and ground-breaking progress in building on this foundation. Researchers have applied the transformer directly to computer vision by slicing images into patches, such as ViT [19] and DETR [20]. The transformer has shown great promise in the field of computer vision, outperforming CNN in various mainstream tasks such as image classification [11] and target detection [12], and is likely to replace CNN as the new backbone in the future, mainly due to the transformer’s ability to capture long-range pixel interactions and aggregate global information from the entire input sequence.

**Figure 1:**
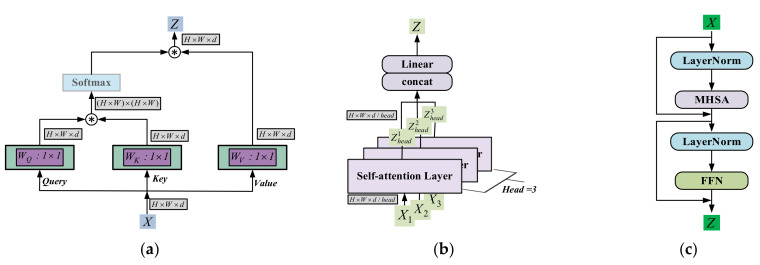
Three key architectures in transformers are depicted schematically. (**a**) Self-Attention; (**b**) Multi-head Self-Attention; (**c**) Transformer encoder.

Transformer mainly uses Multi-Headed Self-Attention (MHSA) to model long-range interactions, as shown in Figure 1a below is the schematic architecture of MHSA. The input image $X_{image}\in {\mathbb{R}}^{H\times W\times C}$ is cropped into $N=HW/P^{2}$ image blocks of size P×P×C, and each image block is expanded into a one-dimensional vector to finally obtain $X\in {\mathbb{R}}^{N\times p^{2}c}$, where *H*, and *W* represent the length and width of the image, respectively, *N* represents the number of image blocks, i.e., the number of tokens, and $d=P^{2}C$ represents the feature dimension of each token. As in Equation (1) *X* is linearly transformed, and together with the class token a new vector is formed as the input to the transformer encoder.

(1) $$X_{input}=[X_{class};X_{1}W;X_{2}W;\cdot \cdot \cdot X_{N}W]+E_{pos}$$

where $X_{class}$ is the class token added to implement the classification task, W is the matrix that implements the linear mapping, and $E_{pos}$ is the position embedding.

Implementing Self-Attention requires defining three key elements. The query $Q=XW_{Q}$, the key $K=XW_{K}$, and the value $V=XW_{V}$. Where $W_{Q},W_{K},W_{V}\in {\mathbb{R}}^{d\times d}$ is the weight matrix that implements the linear mapping. The output *Z* can be formulated as:

(2) $$Z=\mathrm{softmax}(QK^{T}/\sqrt{d})V=\mathrm{Attention}(Q,K,V)$$

where $\sqrt{d}$ represents approximate normalization and the matrix product of $QK^{T}$ calculates the similarity between each pair of tokens to achieve Self-Attention. As shown in Figure 1b, the input *X* is i-quantized, i.e., $X=[x_{1},x_{2},\cdot \cdot \cdot ,x_{i}]$, and $x_{i}\in {\mathbb{R}}^{N\times \frac{d}{i}}$ is entered separately into the Self-Attention module to obtain the multi-headed attention values by the connected single attention values $Z=[z_{1},z_{2},\cdot \cdot \cdot ,z_{i}]$. The formulation can be further rewritten as:

(3) $$Z={\left[\mathrm{Attention}(x_{i}w_{i}^{Q},x_{i}w_{i}^{K},x_{i}w_{i}^{V})\right]}_{1:i}W^{o}$$

where Attention(⋅) is the standard self-attentive function based on QKV, ${\left[\cdot \right]}_{1:i}$ indicating that the results obtained are concatenated.

**Figure 2:**
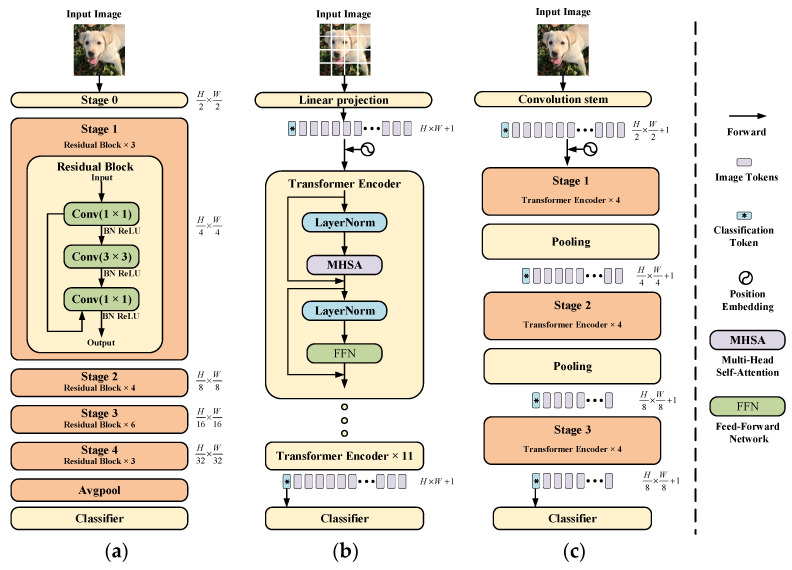
Comparison of three different neural network architectures (**a**) ResNet-50 [21] (**b**) Vision transformer [19] (**c**) Multi-stage transformer [22].

Although the transformer has made breakthroughs in computer vision tasks, there is still a significant performance and computational cost gap between the simple multilayer transformer encoder architecture and the previous convolutional neural network, making the transformer unavailable on limited hardware resources. Figure 2 shows an overview of the ResNet50 [21], the vision transformer [19], and the multi-stage transformer [22]. The main idea of ResNet50 is to divide the feature map extraction into different stages, so that, given an input image, we can generate feature maps at different scales, which has proved to be very useful in many intensive prediction tasks [21,23]. The image is split directly into non-overlapping patches in the vision transformer and then fed into the transformer encoder after linear projection. Inspired by the two architectures mentioned above, The researchers designed a novel transformer network architecture to generate hierarchical feature representations like ResNet50, which usually apply a pooling layer before each stage to reduce the size of intermediate features by 2x downsampling, and stack several encoder blocks in each stage. Compared to traditional vision transformer architecture, this multi-stage transformer network can significantly reduce the number of parameters, allowing us to train and extract multi-scale feature representations that have been shown to be beneficial for many vision tasks. We can obtain four hierarchical feature maps at different resolutions, similar to a typical convolutional neural network. Our proposed Conv-Former network also exploits the advantages of this hierarchical architecture and improves upon it.

**Figure 3:**
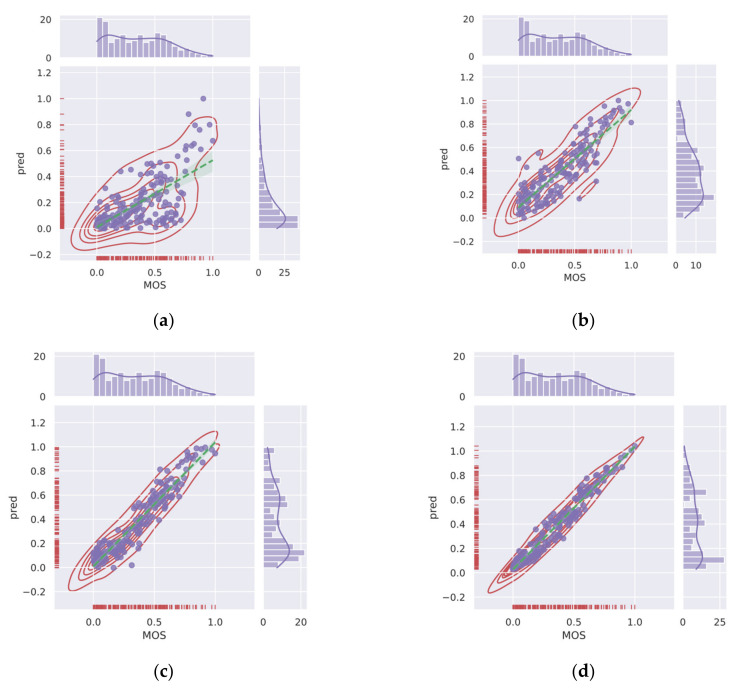
Representative traditional IQA methods and deep learning-based methods are compared with our proposed method on dataset TID2013. Higher correlation means better performance of the IQA method. (**a**) BRISQUE (**b**) NIQE (**c**) DBCNN (**d**) ours.

In this paper, we aim to design an IQA model (Conv-Former) using the long-range interaction capabilities of a transformer and the local feature extraction capabilities of CNN. The network can give predictions that are more consistent with human visual system perception. Therefore, we introduce a multi-stage transformer network architecture to our IQA model. Using a local information perception module and a transformer to capture local information-aware features and global semantic features in an image, the network is able to collect fine-grained detail and global information using both local and global features. The dual-path pooling allows the multi-stage transformer to capture as much contextual information as possible, and the Conv-Former has experimentally proven to be highly capable of local feature perception and image content understanding, both of which are quite important in IQA tasks. The main contributions of this paper are as follows:

(1) We designed an end-to-end neural network model called Conv-Former for no-reference image quality assessment. The overall architecture uses a multi-stage architecture similar to that of ResNet-50 to obtain multi-scale features, which can significantly reduce the number of parameters compared to the traditional transformer architecture. At the same time, multi-scale features are more conducive to the extraction of image quality features. This architecture enables the generation of appropriate perceptual mechanisms in image quality assessment to build an accurate IQA model;
(2) In this work, we introduce an effective hybrid architecture for image quality assessment networks that utilize local information from CNNs and global semantic information captured by the transformer to further improve the accuracy of IQA, implemented by replacing the linear layer that generates the qkv matrix with a local information-aware module that is able to further obtain local information in image quality, acquire fine-grained features and obtain detailed and overall information representation in the image. Network analysis experiments also demonstrate that the network outperforms other models for understanding the content of the input images. This enables the neural network to focus better on the subtle differences between images and thus obtain a more accurate image quality score. In order to reduce the image quality information loss in the feature downsampling process under multi-stage architecture, we designed the dual path pooling module to keep more contextual information;
(3) The position embedding of traditional transformer networks cannot adapt to the input of different resolution images and the use of local information perception modules. Therefore, this paper proposes an adaptive 2D position embedding module, which solves the problem that traditional CNN networks cannot input images with different resolutions, and at the same time, the 2D position embedding is more in line with the characteristics of images. It can effectively represent the position information between tokens;
(4) We experimented with Conv-Former on two different authentic image quality assessment datasets, LIVE Challenge (LIVEC) and KonIQ-10k, as well as on synthetic datasets LIVE, TID2013, and CSIQ, and compared the performance of the algorithm on different distortion types. The extensive experimental results show that Conv-Former has competitive results, which demonstrate the strong fitting performance and generalization capability of our proposed model. As shown in Figure 3, we can find that the results of Conv-Former are more in line with the Mean Opinion Score (MOS).

## 2. Related Work

### 2.1. Attention Mechanism in CNN

The attention mechanism is an important feature of the human visual system, which means that only a portion of all visible information is noticed by humans. The attention mechanism is introduced in CNNs by simulating the human visual perception process, which can ignore the interference of irrelevant information and thus improve the generalization performance of the network. This has enabled CNNs to make breakthroughs in areas such as object detection, image generation, and target tracking.

The attention mechanism was originally used to encode long input sentences as part of the encoder-decoder framework in recurrent neural networks (RNN) and has since been widely used in RNN [24]. Attention is widely used to enhance the representation of features. For example, Hu et al. [25] propose that SENet uses channel attention to explicitly model the interdependencies between feature maps and adaptively acquire the importance of each feature map by learning and then updating the original data based on this importance. In this way, SENet increases the importance of features that are more useful for the task and decreases the importance of useless features to achieve better results. By embedding this module into other networks, the computational resources of the neural network can be more rationally allocated with a small increase in the cost of the number of parameters, resulting in a significant improvement in network performance. Wang et al. [26] proposed efficient channel attention by improving the SENet, which is a local cross-channel interaction strategy without dimensionality reduction and an adaptive selection of the one-dimensional convolutional kernel size to obtain more accurate attention information by aggregating cross-channel information through a one-dimensional convolutional layer. Convolutional Block Attention Module (CBAM) [27] is constructed by combining the spatial attention module (SAM) and the channel. The CBAM is built by combining the spatial attention module (SAM) and the channel attention module (CAM), aggregating attention information from both spatial and channel aspects respectively, and fusing the information to a certain extent to obtain more comprehensive and reliable attention information and provide more appropriate guidance on the allocation of computational resources. Based on CBAM, fu et al. [28] proposed DA-Net, which also integrates channel attention and spatial attention. Unlike CBAM, where the acquisition of attention information in both directions is parallel, DA-Net captures global feature dependencies in the spatial and channel dimensions, using a spatial attention module to learn spatial interdependencies of features and a channel attention module to model channel interdependencies.

### 2.2. No-Reference Image Quality Assessment

No-reference image quality assessment means that no reference image is required, and the quality is assessed only based on the distorted image’s characteristics, which is also more in line with practical needs, as reference images are difficult to obtain or do not exist in practical applications. NR-IQA methods can be divided into two categories, distortion type specific IQA methods [29,30,31] and generic IQA methods [8,9,10]. Distortion type specific IQA methods are designed based on specific distortion types, such as noise, JPEG compression artifacts, blurred artifacts, and other distortion types, and these methods design specific feature extraction methods by looking at the histogram of the pixel distribution of the image after distortion, after which the quality prediction score of the image is obtained. However, this method is limited in that it can only detect quality losses caused by specific distortions and is therefore not widely used. The generic NR-IQA method is more effective because the image distortion type is usually unknown in advance.

**Figure 4:**
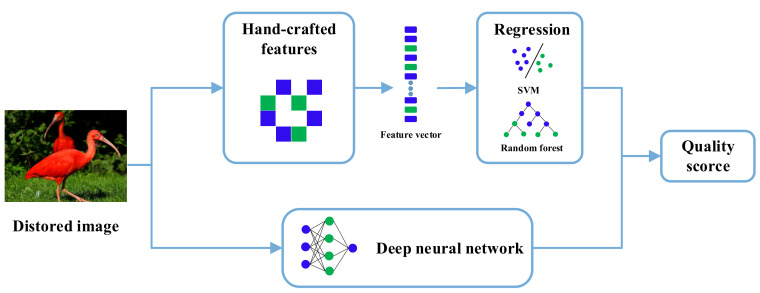
The general process of traditional no-reference image quality assessment methods.

As shown in Figure 4, most of the traditional NR-IQA methods are based on natural scene statistics (NSS) methods, which first manually extract features from distorted images and then use probabilistic or regression models for quality prediction of distorted images. In early NSS-based NR-IQA methods, features are extracted from transform domains such as wavelet or cosine transform domains.

Moorth et al. [32,33] proposed a class of image quality evaluation methods divided into two stages, first identifying distortion types and then performing the distortion-specific quality assessment. For example, Blind Image Quality Index (BIQI) [32], an image authenticity and integrity image quality evaluation algorithm based on distortion type identification (DIIVINE) [33], both of which are based on training a support vector machine (SVM) to obtain a classifier for the image distortion type, then extracting the image features and relying on a support vector regressor to regress the quality prediction scores for each DIIVINE improves the process of extracting image features based on BIQI, uses NSS to estimate the coefficient distribution of wavelets, and extracts global features to determine the image quality score. Saad et al. proposed a blind image integrity labeling algorithm based on discrete cosine transform statistics (BLIINDS) [34], which extracts features from the DCT domain and then uses a multivariate Gaussian model to obtain the quality scores of distorted images. Saad et al. later proposed an optimized BLIINDS (BLIINDS-II) [35] by extracting more complex DCT features, using a generalized Gaussian mixture model to fit different multiscale discrete cosine transform coefficient distributions as frequency domain statistics and a Bayesian which wasmore time-consuming because of the transformation of the image domain. To avoid the transformation of the domain, methods based on spatial domain features have emerged. Mittal et al. [8] proposed a spatial domain non-reference image quality assessment algorithm (BRISQUE), which uses the local mean and variance of the image to calculate the local normalized brightness of the image, and then uses a generalized Gaussian model to model the local normalized brightness distribution as its spatial domain natural statistical features to obtain the prediction score. Ye et al. [36] proposed a codebook-based manual feature extraction NR-IQA algorithm, which uses a K-mean clustering method to learn codebooks directly from training image blocks, then codebooks are used to encode on test images to obtain features of the images, and finally, SVR is used to predict the quality scores of distorted images. Zhang et al. [37] used these features to extract salient regions of semantic objects for quality estimation. Xu et al. [9] improved the feature set and predicted the quality score by merging the higher-order statistical information of the images. However, these manual feature extraction methods require specialized design and are very time-consuming. In addition, scene statistical features characterize image quality from a global perspective and thus cannot measure local distortions common in real distorted images.

Inspired by the breakthroughs in deep learning for other vision tasks [11,12,13,21], researchers have proposed several learning-based methods for image quality assessment that extract quality-related image features and automatically learn correction parameters through deep learning. Thus, better results than traditional manual feature extraction are obtained.

## 3. Proposed Method

In this section, we describe in detail the architecture of our proposed Conv-Former model and outline the specific roles each block plays. First, we describe the overview of the Conv-Former block. On this basis, the key modules of the algorithm are described, such as the local information-awareness module and the adaptive position embedding.

### 3.1. Overview

**Figure 5:**
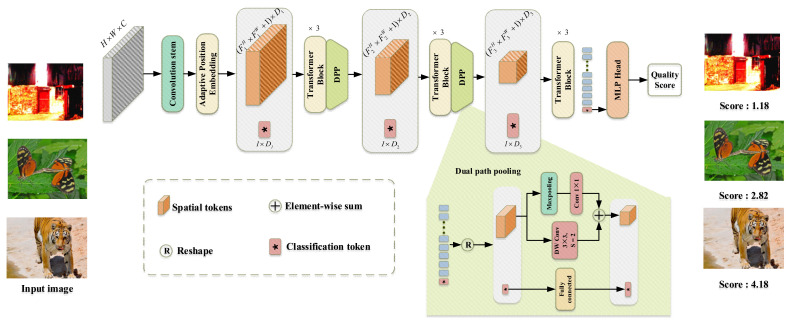
The overview of the network architecture of the proposed Conv-Former.

An overview of the model is depicted in Figure 5. The processing of the input image can be divided into three stages and let the output features of each stage be $F_{1}$, $F_{2}$ and $F_{3}$ respectively. Each feature consists of spatial tokens $\left[X_{spatial}^{1},\cdot \cdot \cdot ,X_{spatial}^{3}\right]$ and classification token $\left[X_{class}^{1},\cdot \cdot \cdot ,X_{class}^{3}\right]$ and can be expressed by Equation (4). In this paper, the feature channel numbers *D1*, *D2*, and *D3* are taken to be 192, 384, and 512 respectively.

(4) $$\begin{array}{l} F_{1}=[X_{class}^{1};X_{spatial}^{1}],X_{class}^{1}\in {\mathbb{R}}^{1\times D_{1}},X_{spatial}^{1}\\ F_{2}=[X_{class}^{2};X_{spatial}^{2}],X_{class}^{2}\in {\mathbb{R}}^{1\times D_{2}},X_{spatial}^{2}\\ F_{3}=[X_{class}^{3};X_{spatial}^{3}],X_{class}^{3}\in {\mathbb{R}}^{1\times D_{3}},X_{spatial}^{3} \end{array}$$

First, we reshape the input image $X\in {\mathbb{R}}^{H\times W\times C}$ to the feature map $F_{1}\in {\mathbb{R}}^{\frac{H}{p}\times \frac{W}{p}\times D_{1}}$ by convolutional feature extraction, where (*H*, *W*) is the resolution of the original image, *C* is the number of channels, *p* is the resolution decay after the convolution operation, $D_{1}$ is the dimension of the feature map $F_{1}$, and $N=HW/P^{2}$ is the resulting number of tokens, which serves as the effective input sequence length for the Transformer. The Adaptive Position Embedding described in Section 3.2 is added to the patch embeddings to retain positional information. As shown in Equation (6), The process from feature $F_{1}$ to $F_{2}$ is similar to the process from $F_{2}$ to $F_{3}$, with three layers of transformer modules and a layer of Dual path pooling(DDP) modules for 2x down adoption in between, reducing the resolution of the features while increasing the number of feature channels, and after obtaining feature $F_{3}$, the classification token containing the image quality information is fed alone into the MLP to obtain the final image quality assessment score.

(5) $$\begin{array}{l} X\to F_{1}=[X_{class}^{1};Conv(X)+E_{pos}],E_{pos}\in {\mathbb{R}}^{F_{1}^{H}\times F_{1}^{W}},X_{class}^{1}\in {\mathbb{R}}^{1\times D_{1}}\\ F_{1}\to F_{2}=DPP(TB(TB(TB(F_{1}))))\\ F_{2}\to F_{3}=DPP(TB(TB(TB(F_{2}))))\\ F_{3}\to Score=MLP(X_{class}^{3}),X_{class}^{3}\in {\mathbb{R}}^{1\times D_{3}} \end{array}$$

### 3.2. Adaptive 2D Position Embedding

In CNN-based image quality evaluation models, the input images need to be resized or cropped to a fixed shape for batch training. However, this pre-processing changes the aspect ratio and composition of the image, which affects the image quality. However, by processing the position encoding part of the transformer-based network, it is possible to input images of any resolution into the network. No pre-processing of the input image is required, in line with the human visual system.

Position encoding is an integral part of the transformer architecture, through which the position encoding can be determined to explicitly model the position of the token and improve the representational power of the model. Its effectiveness has been well demonstrated in the field of natural language processing [15,16,18]. Since images can be considered as two-dimensional sequences, there is a need to extend the one-dimensional position encoding to two-dimensional position encoding, regardless of the input image size. The method mentioned in this section can effectively provide the position information required for object localization.

**Figure 6:**
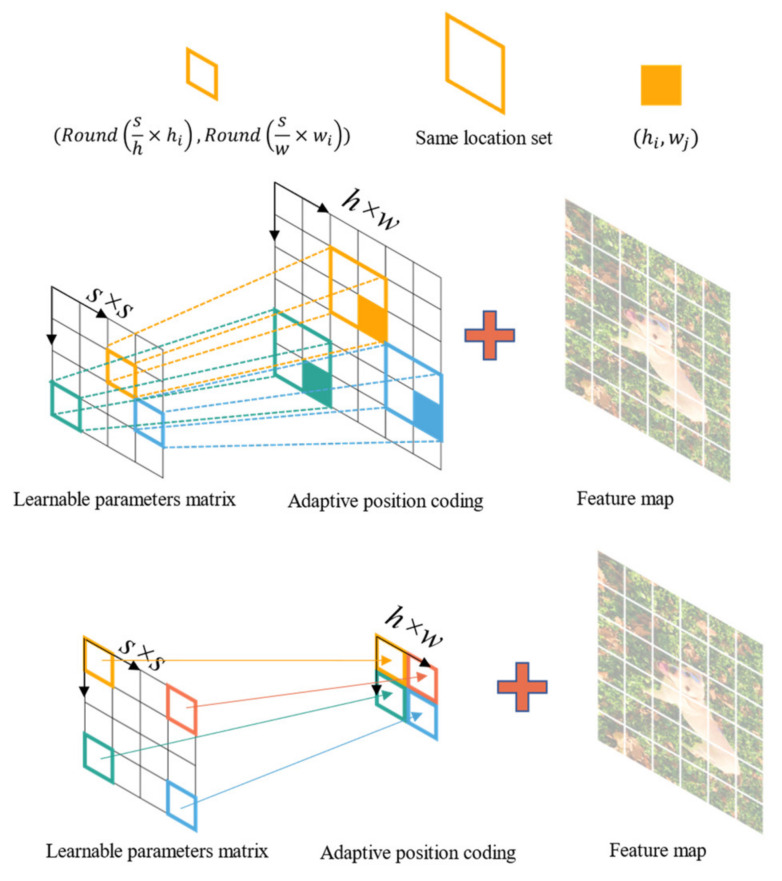
Schematic drawing of the implementation of adaptive 2D position embedding.

The specific implementation steps are as follows. Suppose the size of the input feature map is ${\mathbb{R}}^{h\times w\times c}$, then we define a learnable parameter matrix $L\in {\mathbb{R}}^{s\times s}$, where the size of the matrix *s* is a hyperparameter, set to 10 in this paper, and we obtain the position code $A\in {\mathbb{R}}^{h\times w}$ by adaptively deflating the learnable matrix, the size of the position code is consistent with the feature map, as shown in Figure 6, let $\left(h_{i},w_{j}\right)$ be a point on the position code matrix A, then the corresponding position code at that point can be determined by the following equation.

(6) $$A(h_{i},w_{j})=L(Round(\frac{s}{h}\times h_{i}),Round(\frac{s}{w}\times w_{i}))$$

where Round(⋅) stands for rounding the floating point number inside the brackets and A(⋅ , ⋅), L(⋅ , ⋅), represent the coded values at the corresponding positions respectively.

### 3.3. Transformer Block

**Figure 7:**
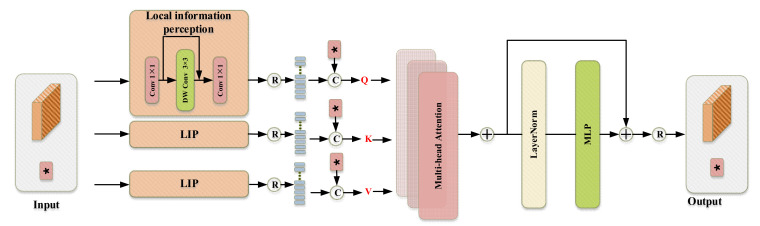
The architecture of the proposed Transformer Block.

Local features can be captured in CNN by convolutional operations, and although global features can be captured by continuously deepening the neural network, the global features suffer a significant loss in the process. With the advent of transformer, the long-range dependencies of token are captured by Self-Attention and multi-layer perceptron (MLP) architecture, but such architectures ignore local detail features. As shown in Figure 7, in order to combine the advantages of local features and global representations and thus improve the performance of the transformer network, we designed a novel local information perception (LIP) module to generate QKV that improves the discriminability between background and foreground. As shown in Equation (7), Let the input tensor $X\in {\mathbb{R}}^{h\times w\times d}$ be projected into the query vector $Q\in {\mathbb{R}}^{L\times d}$, key vector $K\in {\mathbb{R}}^{L\times d}$, and value tensor $V\in {\mathbb{R}}^{L\times d}$, where *d* is the dimension size of each token and L=h×w+1 is the number of tokens.

(7) $$\left\{\begin{array}{c} Q=concate[flatten(LIP_{Q}(X)),T_{cls}]\\ K=concate[flatten(LIP_{K}(X)),T_{cls}]\\ V=concate[flatten(LIP_{V}(X)),T_{cls}] \end{array}\right.$$

where $T_{cls}$ stands for classification token.

(8) LIP(X)=Conv(Conv(x)+DWConv(x))

### 3.4. Dual Path Pooling

For the feature downsampling at the end of each stage, we designed a dual-path pooling (DPP) layer, as shown in Figure 5. It consists of two branches: one is a 3 × 3 depthwise convolution with a step size of two; the other is a pooling layer and a 1 × 1 convolution. It is possible to achieve twice as much downsampling. During feature downsampling, the features on both paths are fused together by channel stacking to retain more contextual information. Experimental results show that DPP performs better than a direct maximum pooling layer. In equation terms, this can be described as follows.

(9) I↓=concat(DWconv(x,3×3)+conv(maxpooling(x),1×1))

## 4. Experiments

### 4.1. Datasets

In this work, five widely used datasets in the field of image quality assessment were used, which can be split into authentic datasets and synthetic datasets based on the method of obtaining distorted images. The synthetic datasets include LIVE [38], TID2013 [39], and CSIQ [40]. The authentic distortion image dataset includes the LIVE Challenge (LIVEC) [41] and KonIQ-10k [42] datasets. A detailed description of them is given in Table 1.

The University of Texas at Austin’s Image and Video Engineering Laboratory established the LIVE image quality assessment dataset [38] in 2006. It consists of 779 distorted images developed from 29 source images using a total of five different forms of distortion (JP2K compression, JPEG compression, additive white Gaussian noise, Gaussian blur, and Simulated fast-fading Rayleigh channel). The scores are expressed by the Differential Mean Opinion Score (DMOS), the difference between the human eye’s evaluation score of the reference image and the distorted image, with lower values indicating higher image visual quality.

**Figure 8:**
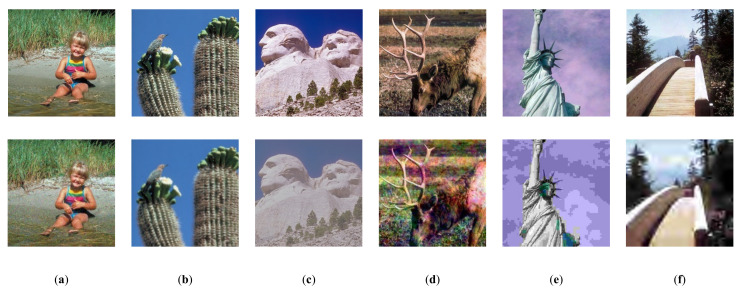
Distorted images in the synthetic dataset CSIQ and the corresponding reference images, corresponding to the distortion types (**a**) White Noise (**b**) Gaussian Blur (**c**) Contrast stretching (**d**) Pink noise (**e**) JPEG Compression (**f**) JPEG2000 Compression.

The TID2013 dataset is an extension of the TID2008 dataset [43] and contains 3000 distorted images based on 25 reference images with 24 different distortion types and five distortion levels. Image distortion categories include Additive Gaussian noise, Impulse noise, Chromatic aberrations, and so on. The Mean Opinion Score (MOS) values [0, 9] are employed. The higher the value, the greater the visual quality. Because the TID2013 dataset contains more types of distortion, it places more demands on the algorithm, and many traditional methods cannot be used effectively. In Figure 8, we compare the attention maps of the three different network architectures.

**Figure 9:**
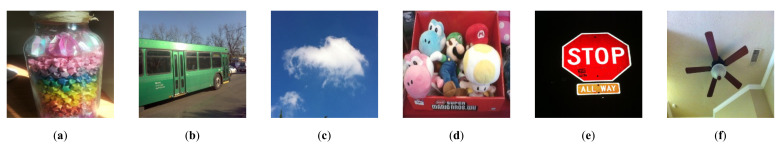
(**a**–**f**) are the distorted images from the authentic dataset LIVE Challenge.

The Computational Perception and Image Quality Lab at Oklahoma State University created the CSIQ dataset [40], which contains 30 raw images and 866 images distorted by JPEG compression, JP2K compression, Gaussian blur, Gaussian white noise, Gaussian pink noise, or contrast variation, with five or four levels of each distortion type. The photos are 512 × 512 in size. The DMOS values acquired are in the [0, 1] range, with lower values suggesting greater visual quality. We show a selection of images from the dataset in Figure 9.

**Figure 10:**
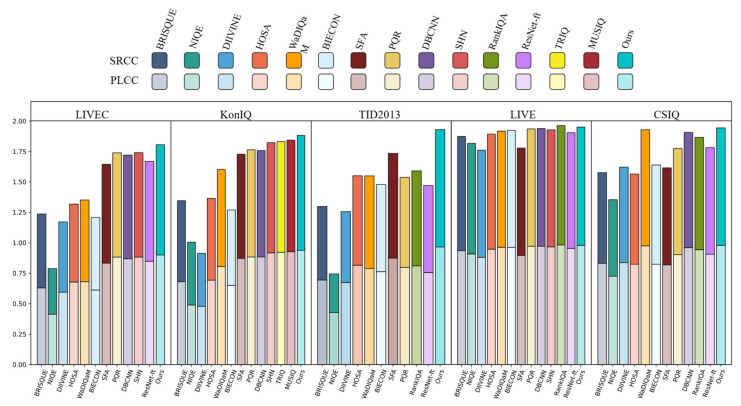
Histogram representation of the data in Table 2.

LIVE Challenge [41] contains 1162 images taken in a variety of natural environments, with complex losses due to the level of photography and imaging equipment used to capture them, typically a combination of overexposure or underexposure, blur, grain, or compression, with MOS ranging from [0, 100], the higher the value the better. We show a selection of images from the dataset in Figure 10.

The KonIQ-10k dataset consists of 10,073 images selected from the large public multimedia database YFCC100m [44]. The sampled images cover as wide and uniform a quality distribution as possible in terms of brightness, colour, contrast and sharpness, and the types of distortion present in these images include noise, JPEG artifacts, blending, lens motion blur, over-sharpening, and so on. The researchers conducted a large-scale crowdsourcing experiment based on the collected dataset, receiving 1.2 million assessments from 1467 observers utilizing statistical approaches such as taking the mean and deleting extreme scores to determine the final MOS values. The photos were 1024 × 768 in size. MOS values were in the [0, 5] range, with higher values indicating less distortion.

### 4.2. Evaluation Metrics

In order to quantitatively compare the performance of IQA algorithms, researchers often use the following three evaluation criteria.

(1) Spearman rank-order correlation coefficient (SROCC), SRCC is used to measure the monotonicity of IQA algorithm predictions and is calculated as follows.

(10) $$\mathrm{SRCC}=1-\frac{6\sum_{i}d_{i}^{2}}{I(I^{2}-1)}$$

where $d_{i}$ denotes the difference between the subjective quality score ranking of the *i*-th image and the objective quality score ranking, and *I* denotes the number of images in the test set.

(2) The Pearson linear correlation coefficient (PLCC), PLCC is used to assess the accuracy and degree of linear correlation of IQA model predictions.

(11) $$\mathrm{PLCC}=\frac{\sum_{i}\left(q_{i}-q_{m})({\hat{q}}_{i}-{\hat{q}}_{m}\right)}{\sqrt{\sum_{i}{\left(q_{i}-q_{m}\right)}^{2}}\sqrt{\sum_{i}{\left({\hat{q}}_{i}-{\hat{q}}_{m}\right)}^{2}}}$$

where $q_{i}$ and ${\hat{q}}_{i}$ denote the MOS value and algorithm prediction score of the *i*-th image, respectively, and $q_{m}$ ${\hat{q}}_{m}$ denote the mean MOS value and the mean algorithm prediction score of the test image samples, respectively.

(3) The root mean square error (RMSE), RMSE is used to assess the consistency of the IQA model’s predictions. It is used to measure the absolute error between the algorithm’s predicted score and the subjective evaluation score and is calculated as follows.

(12) $$\mathrm{RMSE}=\sqrt{\frac{1}{n}\sum_{i=1}^{N}{\left(q_{i}-{\hat{q}}_{i}\right)}^{2}}$$

### 4.3. Implementation Details

In the experiments, for each dataset, 80% of the images were randomly selected for training and 20% for testing. The training is conducted using a SGD optimizer with a batch size of eight. We trained our models with an initial learning rate of 0.001, with a warm up cosine learning rate decay scheduler. We adopted MSE loss for training:

(13) $$L_{reg}=∥model(I_{Dist})-s{∥}_{2}$$

where $model(I_{Dist})$ denotes the output of the proposed Conv-Former, *s* denotes the ground-truth normalized MOS or DMOS value. We implemented our proposed model Conv-Former in Pytorch version 1.12.0 and python version 3.9, which was trained using a single NVIDIA GeForce RTX 3090 GPU. The CUDA and CuDNN versions are 11.6 and 8.4.0 respectively.

### 4.4. Comparing with the State-of-The Art (SOTA)

We assessed the performance of our model with PLCC and SRCC. PLCC assesses the linear correlation between ground truth and the predicted quality scores, whereas SRCC describes the level of monotonic correlation.

**Figure 11:**
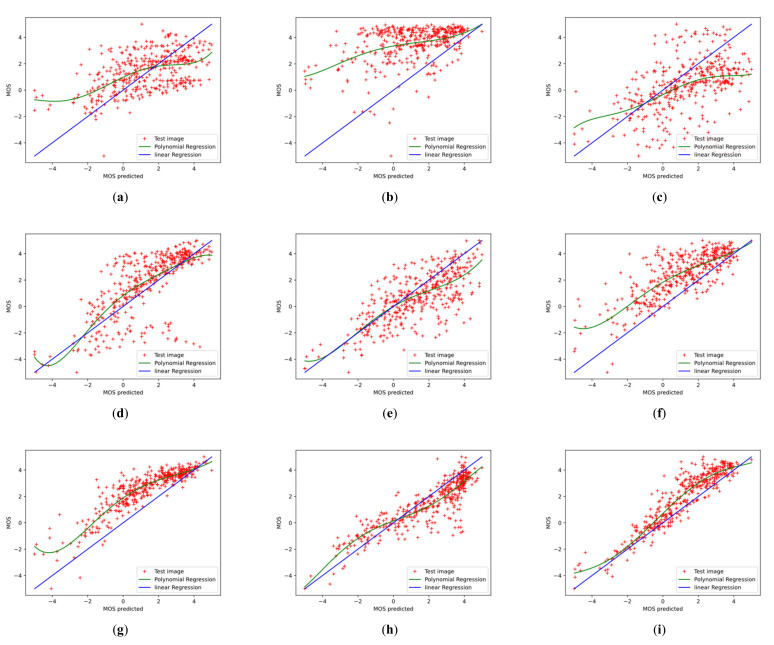
Scatter plots of ground-truth mean opinion scores (MOS) against predicted scores of (**a**–**f**) different IQA algorithms on CSIQ datasets. The red points denote the testing instances. The blue line is the ideal linear relationship between MOS and the prediction score. The green line is the curve fitted using the test instance. Higher correlation means better performance of the IQA method. (**a**) BRISQUE; (**b**) NIQE; (**c**) DIIVINE; (**d**) HOSA; (**e**) CORNIA; (**f**) BIECON; (**g**) SFA; (**h**) WaDIQaM; (**i**) ours.

We evaluated the effectiveness of Conv-Former on five benchmark datasets. For all of our tests, we followed the above experimental setup. It can be shown in Table 2 that Conv-Former outperforms or is competitive with 14 NR-IQA methods: BRISQUE, NIQE, DIIVINE, HOSA, WaDIQaM, BIECON, SFA, PQR, DBCNN, SHN, RankIQA, ResNet-ft, TRIQ and MUSIQ. We found that our method achieves the best PLCC/SRCC results in comparison to other works. Especially on the moe complex dataset TID2013, our proposed model achieved a solid improvement over previous work. Even though Conv-Former achieves 0.965 on PLCC and 0.964 on SRCC, which means the metric is consistent with the human perspective. The effective feature fusion by CNN and ViT and the proposed multi-scale prediction module make our method substantially superior to other transformer-based image quality assessment network; examples include TRIQ and MUSIQ. Although the model achieved better results on more complex datasets, there was no major improvement for datasets where most algorithms performed well, such as the LIVE dataset. In order to visualise the advantages of Conv-Former, we present the data in Table 2 as a histogram in Figure 11.

In order to evaluate the effectiveness of Conv-Former on different types of distorted images, we also compare the PLCC/SRCC performances on five kinds (JP2K compression, JPEG compression, additive white Gaussian noise, Gaussian blur, Simulated fast fading Rayleigh channel) of distorted types in the LIVE dataset and six kinds (JPEG compression, JP2K compression, Gaussian blur, Gaussian white noise, Gaussian pink noise, contrast variation) of distorted types in the CSIQ dataset. Table 3 and Table 4 show the results of different IQA methods on different types of distorted images. In the tables, we find our method achieves the best or most competitive performances on all different types of distorted images than other IQA methods.

**Figure 12:**
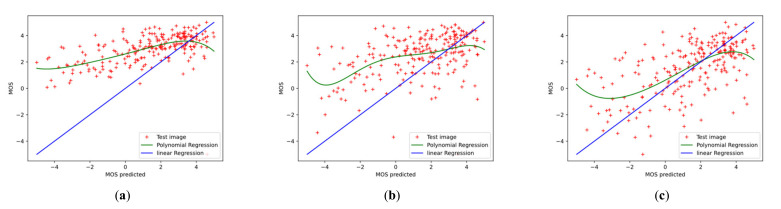

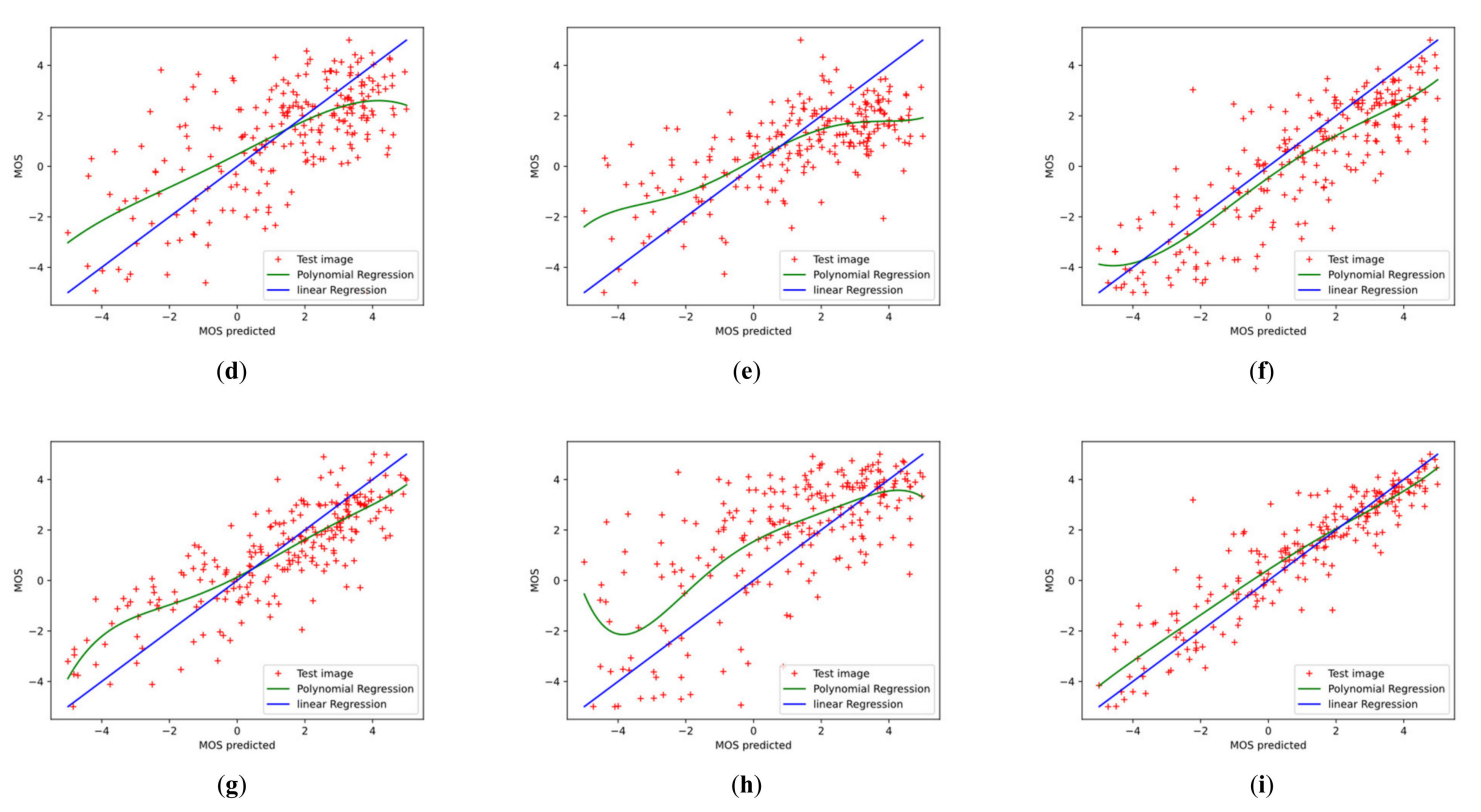
Scatter plots of ground-truth mean opinion scores (MOS) against predicted scores of (**a**–**f**) different algorithms on LIVEC datasets. The red points denote the testing instances. The blue line is the ideal linear relationship between MOS and the prediction score. The green line is the curve fitted using the test instance. Higher correlation means better performance of the IQA method. (**a**) BRISQUE; (**b**) NIQE; (**c**) DIIVINE; (**d**) HOSA; (**e**) CORNIA; (**f**) BIECON; (**g**) SFA; (**h**) WaDIQaM; (**i**) ours.

**Figure 13:**
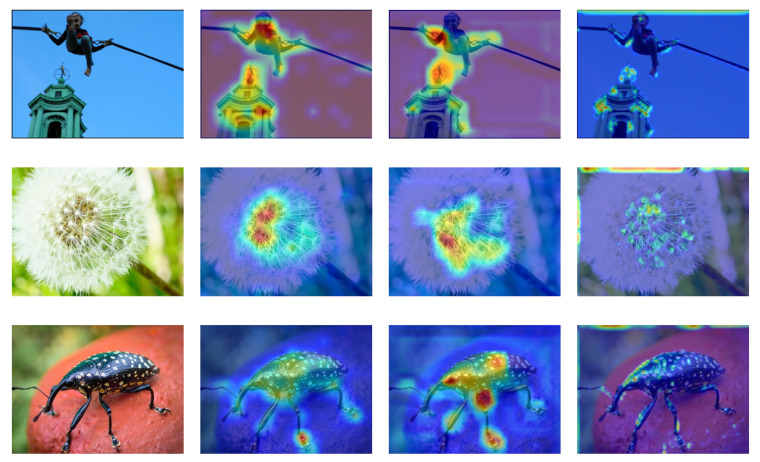

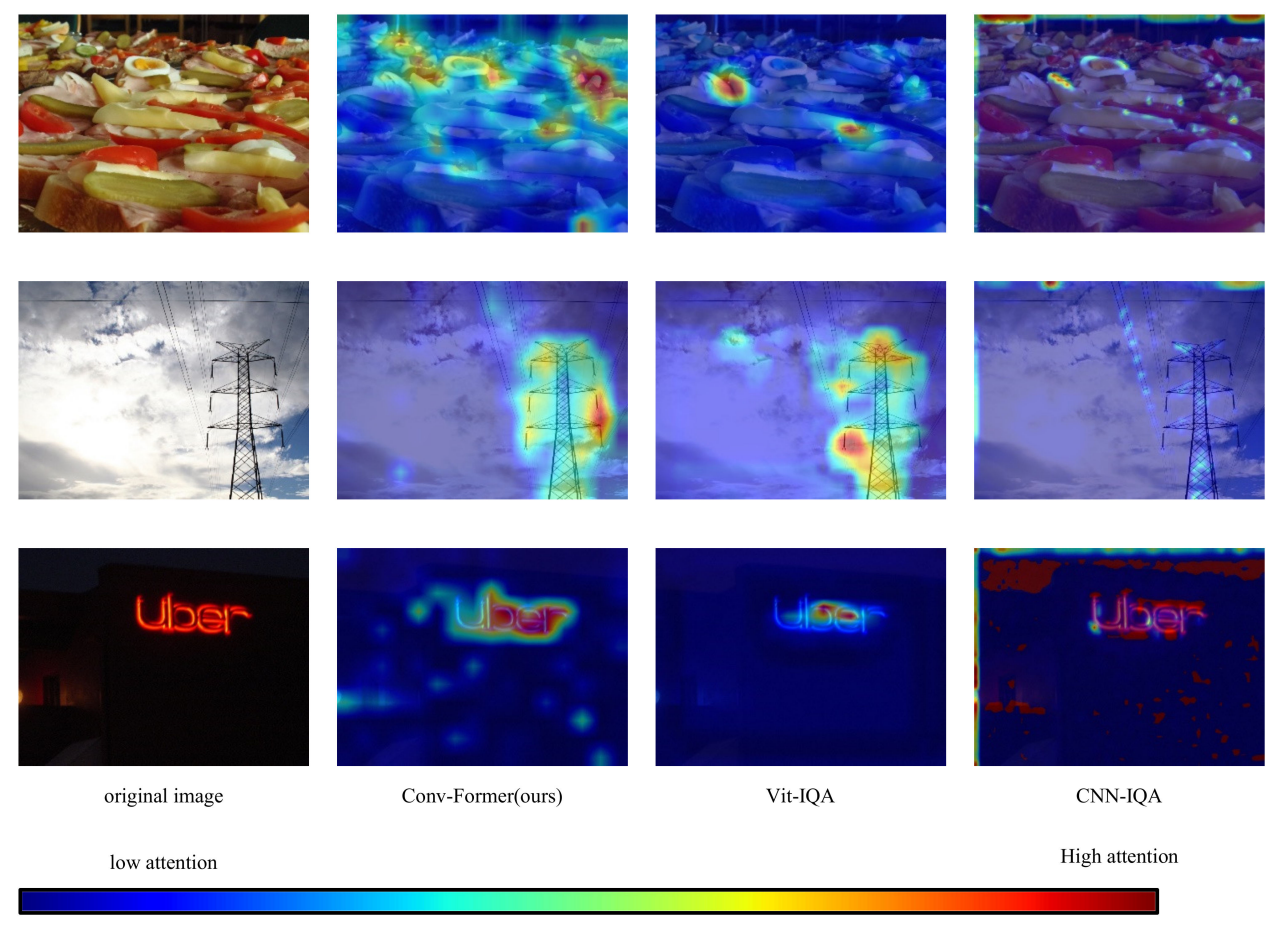
Comparison of attention maps for three different architectures of neural networks. All attentions are normalized in the range 0 to 1. The red areas mean the higher attention values, and the blue areas mean the lower attention values. The visual sensitive areas of our approach are more distinguishable.

To further investigate the effectiveness of the proposed Conv-Former, we demonstrate the scatter plots between the MOS and the prediction scores and analyze the correlation. Figure 12 and Figure 13 show the scatter plots of different IQA methods on the CSIQ and LIVEC datasets, respectively. The red points denote the testing instances. The blue line is the ideal linear relationship between MOS and the prediction score. The green line is the curve fitted using the test instance. All values are normalized in the range of −5 to 5 for a better view. In the Figure 12 and Figure 13, we can find that the results of Conv-Former are more in line with the MOS. Compared with WaDIQaM and BIECON, our Conv-Former has fewer outliers and performs more consistently with the MOS.

To evaluate the generalization of our proposed Conv-Former, we conduct the cross dataset evaluation on LIVE, CSIQ, TID2013, and LIVEC. We train the model on one train dataset separately, then test it on the full set of the other three benchmark datasets. As shown in Table 5, Conv-Former achieves good generalization ability.

### 4.5. Ablation Studies

In this section, to evaluate the efficiency of our proposed components, we analyze the effectiveness of the proposed network by conducting ablation studies. With different configuration and implementation strategies, we evaluate the effect of each of the four major components: Multi-Scale transformer architecture (MS), Adaptive Position Embedding (APE), Dual Path Pooling (DPP), and local information perception (LIP) module. We conduct ablation experiments on the LIVEC, KonIQ, CSIQ, and TID2013 databases. The results are shown in Table 6.

We first examine the effectiveness of our proposed Adaptive Position Embedding module. From the results, we can see a significant improvement in SRCC and PLCC with the use of positional coding. In KonIQ, the SRCC and PLCC increased by 2.8% and 2.2%, respectively. As a result, this demonstrates the critical importance of Position Embedding for our proposed Conv-Former. All subsequent ablation experiments were carried out with the Position Embedding. Table 6 shows that the best performance can be achieved when all three components are available. The table shows that the lack of any of the following three components in our Conv-Former will negatively impact the objective performance metrics.

### 4.6. Analysis and Discussion

To further validate the proposed method’s effectiveness and analyze the internal mechanism of the difference in the performance of the neural networks, in Figure 8, we compare the attention maps of the three different network architectures.

The proposed Conv-Former tends to focus on the regions that significantly impact the quality assessment scores, as seen from the attention maps so that the obtained image quality assessment scores can be remarkably consistent with the subjective assessment results of the human eye. For example, people tend to assess the quality of an image based on the target when it is in a pure background, and some images are blurred to highlight the target, so people tend to focus on the clear parts to assess the quality of the image. This aligns with our intuition, resulting in superior results compared to other methods. Compared to the traditional approach using CNN, the approach using the transformer tends to activate more significant regions rather than local areas, implying enhanced long-range feature dependency. Since the local information-aware module provides detailed local features, the Conv-former can retain important detailed local features that are often corrupted by the vision transformer. In addition, the target attention areas can be more complete in a significant area context, meaning that Conv-former learns feature representations with higher discriminative power.

On the other hand, humans perceive different image qualities in different ways when the image’s content is different. Our proposed Conv-Former can identify the image content well in the process of image quality evaluation and can try to understand the image before predicting it, which is more in line with the laws of human perception of the objective world.

## 5. Conclusions

In this paper, we propose a novel network, combining convolution and Self-Attention for an image quality assessment network (Conv-Former) for the no-reference image quality assessment task. Our model can obtain global features through transformer and local information perception (LIP). We evaluate the effectiveness of Conv-Former on five benchmark datasets, we found that our method achieves the best PLCC/SRCC results compared to other works. Especially on the more complex dataset TID2013, our proposed model achieved a solid improvement over previous work. Even though Conv-Former achieves 0.965 on PLCC and 0.964 on SRCC, which means the metric is consistent with the human perspective. Experimental results showed that our proposed approach outperforms the state-of-the-art (SOTA) methods on IQA databases, which has strong generalization ability and provides prospects for the broader application of IQA tasks. Future work will focus on developing more generic IQA models in which a single model can be adapted to diverse image content and imaging devices. Our proposed model should have a sufficient dataset so that the trained model can have a stronger generalization ability and obtain excellent results on images where the model has not been trained. The size of the model is also a key factor in its ability to be deployed in practical applications, and we will continue to optimize the model in the future so that it has fewer parameters and runs faster.

## Figures and Tables

**Table 1:** IQA datasets for performance evaluation and model training.

Database	Reference Image	Distorted Image	Distorted Type	Score Type	Score Range
LIVE	29	779	5	DMOS *	[0, 100]
TID2013	25	3000	24	MOS	[0, 9]
CSIQ	30	866	6	DMOS	[0, 1]
LIVE Challenge	-	1162	-	MOS	[0, 100]
KonIQ-10k	-	10,073	-	MOS	[0, 5]

* DMOS indicates the difference in mean opinion scores (MOS) between the test image and its reference image.

**Table 2:** Performance comparison of the IQA methods on five standard IQA databases, i.e., LIVEC, KonIQ, TID2013, LIVE, CSIQ. The top-performing method is highlighted in boldface.

Methods	LIVEC		KonIQ		TID2013		LIVE		CSIQ	
	PLCC	SRCC	PLCC	SRCC	PLCC	SRCC	PLCC	SRCC	PLCC	SRCC
BRISQUE	0.629	0.608	0.681	0.665	0.694	0.604	0.935	0.939	0.829	0.746
NIQE	0.415	0.373	0.489	0.516	0.426	0.317	0.908	0.908	0.725	0.627
DIIVINE	0.595	0.576	0.479	0.434	0.672	0.583	0.881	0.879	0.836	0.784
HOSA	0.678	0.640	0.694	0.671	0.815	0.735	0.947	0.946	0.823	0.741
WaDIQaM	0.680	0.671	0.805	0.797	0.787	0.761	0.963	0.954	0.973	0.955
BIECON	0.613	0.595	0.651	0.618	0.762	0.717	0.962	0.961	0.823	0.815
SFA	0.833	0.812	0.872	0.856	0.873	0.861	0.895	0.883	0.818	0.796
PQR	0.882	0.857	0.884	0.880	0.798	0.739	0.971	0.965	0.901	0.873
DBCNN	0.869	0.851	0.884	0.875	-	-	0.971	0.968	0.959	0.946
SHN	0.882	0.859	0.917	0.906	-	-	0.966	0.962	0.942	0.923
RankIQA	- *	-	-	-	0.810	0.780	**0.982**	**0.981**	-	-
ResNet-ft	0.849	0.819	-	-	0.756	0.712	0.954	0.950	0.905	0.876
TRIQ	-	-	0.922	0.910	-	-	-	-	-	-
MUSIQ	-	-	0.928	0.916	-	-	-	-	-	-
Ours	**0.899**	**0.868**	**0.938**	**0.924**	**0.965**	**0.964**	0.979	0.971	**0.978**	**0.964**

* indicates that no testing was undertaken on this dataset in the original.

**Table 3:** Average PLCC and SRCC results of individual distortion types on the LIVE databases. The top-performing method is highlighted in boldface.

	Methods	JPEG	JPEG200	WN	GB	FF
PLCC	BRISQUE	0.971	0.940	0.989	0.965	0.894
	HOSA	0.967	0.949	0.983	0.967	0.967
	CORNIA	0.962	0.944	0.974	0.961	0.943
	DBCNN	0.986	0.967	0.988	0.956	0.961
	Ours	**0.987**	**0.977**	0.986	**0.974**	**0.970**
SRCC	BRISQUE	0.965	0.929	**0.982**	**0.966**	0.828
	HOSA	0.954	0.935	0.975	0.954	0.954
	CORNIA	0.947	0.924	0.958	0.951	0.921
	DBCNN	0.972	0.955	0.980	0.935	0.930
	Ours	**0.974**	**0.960**	0.971	0.965	**0.965**

**Table 4:** Average PLCC and SRCC results of individual distortion types on the CSIQ databases. The top performing method is highlighted in bold face.

	Methods	JPEG	JPEG200	WN	GB	PN	CC
PLCC	BRISQUE	0.828	0.887	0.742	0.891	0.496	0.835
	HOSA	0.759	0.899	0.656	0.912	0.601	0.744
	CORNIA	0.563	0.883	0.687	0.904	0.632	0.543
	DBCNN	**0.982**	0.971	0.956	0.969	0.950	0.895
	MEON	0.979	0.925	**0.958**	0.946	-	-
	Ours	0.966	**0.987**	0.951	**0.976**	**0.982**	**0.947**
SRCC	BRISQUE	0.806	0.840	0.723	0.820	0.378	0.804
	HOSA	0.733	0.818	0.604	0.841	0.500	0.716
	CORNIA	0.513	0.831	0.664	0.836	0.493	0.462
	DBCNN	0.940	0.953	0.948	**0.947**	0.940	0.870
	MEON	0.948	0.898	0.951	0.918	-	-
	Ours	**0.969**	**0.980**	**0.975**	0.945	**0.965**	**0.924**

**Table 5:** SRCC evaluations on cross database tests. The top-performing method is highlighted in boldface.

**Train**	**Test**	**Methods**						
		**BRISQUE**	**M3**	**FRIQUEE**	**CORNIA**	**HOSA**	**DB-CNN**	**Ours**
**LIVE**	CSIQ	0.562	0.621	0.722	0.649	0.594	0.758	**0.762**
	TID2013	0.358	0.344	0.461	0.360	0.361	0.524	**0.563**
	LIVEC	0.337	0.226	0.411	0.443	0.463	0.567	**0.572**
CSIQ	LIVE	0.847	0.797	0.879	0.853	0.773	**0.877**	0.864
	TID2013	0.454	0.328	0.463	0.312	0.329	0.540	**0.572**
	LIVEC	0.131	0.183	0.264	0.393	0.291	0.452	**0.463**
TID2013	LIVE	0.790	0.873	0.755	0.846	0.846	0.891	**0.894**
	CSIQ	0.590	0.605	0.635	0.672	0.612	0.807	**0.853**
	LIVEC	0.254	0.112	0.181	0.293	0.319	0.457	**0.524**
LIVEC	LIVE	0.238	0.059	0.644	0.588	0.537	0.746	**0.752**
	CSIQ	0.241	0.109	0.592	0.446	0.336	0.697	**0.711**
	TID2013	0.280	0.058	0.424	0.403	0.399	**0.424**	0.417

**Table 6:** PLCC and SRCC results of ablation experiments on the LIVEC, KonIQ, CSIQ, and TID2013 database.

Methods				LIVEC		KonIQ		CSIQ		TID2013	
APE	MS	LIP	DPP	PLCC	SRCC	PLCC	SRCC	PLCC	SRCC	PLCC	SRCC
×	✓	✓	✓	0.871	0.856	0.922	0.913	0.972	0.953	0.958	0.945
✓	×	✓	✓	0.893	0.866	0.925	0.916	0.973	0.960	0.959	0.950
✓	✓	×	✓	0.881	0.860	0.928	0.921	0.970	0.961	0.955	0.948
✓	✓	✓	×	0.890	0.862	0.933	0.923	0.975	0.963	0.963	0.960
✓	✓	×	×	0.882	0.858	0.930	0.921	0.969	0.956	0.961	0.950
✓	×	✓	×	0.894	0.859	0.924	0.914	0.972	0.961	0.960	0.952
✓	×	×	✓	0.878	0.854	0.929	0.918	0.965	0.953	0.949	0.938
✓	✓	✓	✓	**0.899**	**0.868**	**0.938**	**0.924**	**0.978**	**0.964**	**0.965**	**0.964**

“✓” means that Conv-Former contains this component, “×” means that Conv-Former does not contain this component.

## Data Availability

Not applicable.
